# Editorial: ESKAPEing host endogenous and exogenous defenses: new insights into the ability of multidrug- resistant bacteria to overcome host defenses and antimicrobial compounds activity

**DOI:** 10.3389/fcimb.2026.1837229

**Published:** 2026-04-14

**Authors:** Fiorentina Ascenzioni, Luca Cavinato, Nayeli Alva-Murillo

**Affiliations:** 1 Department of Biology and Biotechnology “Charles Darwin”, Sapienza University, Rome, Italy; 2 Department of Molecular Microbiology, Washington University School of Medicine, St. Louis, MO, United States; 3 Department of Biology, University of Guanajuato, Guanajuato, Mexico

**Keywords:** alternative antimicrobial strategies, antimicrobial resistance mechanisms, ESKAPE bacteria, host-pathogen interactions, multidrug-resistance (MDR)

The global rise of multidrug-resistant (MDR) pathogens constitutes a major public health concern, with a substantial proportion of originating from nosocomial settings (Muhammad et al., 2026). The ESKAPE pathogens (*Enterococcus faecium*, *Staphylococcus aureus*, *Klebsiella pneumoniae*, *Acinetobacter baumannii*, *Pseudomonas aeruginosa*, and *Enterobacter* spp.) are WHO priority bacteria characterized by their capacity to evade antimicrobial activity (De Oliveira et al., 2020). These pathogens are responsible for severe, persistent infections, particularly in immunocompromised individuals, due to their ability to withstand and subvert host immune defenses (Yu et al., 2024). Their persistence relies on multiple antimicrobial resistance mechanisms, including antibiotic target modification, enzymatic drug inactivation, reduced intracellular accumulation, and biofilm formation, which complicate therapeutic eradication (Sakalauskienė et al., 2025).

In response to the diminishing effectiveness of conventional antibiotics, alternative strategies, including host-directed therapies, anti-virulence approaches, and bacteriophage interventions, have gained increasing interest. This Research Topic aims to elucidate the mechanisms by which ESKAPE pathogens overcome endogenous and exogenous host defenses while exploring innovative therapeutic avenues. Comprising four 4 original research articles, this open-access Research Topic provides cutting-edge evidence aimed at expanding current knowledge and advancing alternative treatment options against ESKAPE pathogens.

*K. pneumoniae* is an opportunistic Gram-negative pathogen responsible for diverse healthcare-associated infections worldwide, some of which represent a growing concern in oncology settings (Abbas et al., 2024; Nham et al., 2020). Among them, carbapenem-resistant *K. pneumoniae* (CRKP) infections constitute a high priority threat given their rapid nosocomial dissemination, scarce therapies, and significant mortality burden (Melinte et al., 2025). *K. pneumonia* can be classified as hypervirulent (hvKP) and classical (cKP) according to its virulence level (Chen et al., 2025). In this sense, carbapenem-resistant hypervirulent *K. pneumoniae* (CR-hvKP) has emerged as a critical public health concern driven by the convergence of hypervirulence and antimicrobial resistance (AMR), largely facilitated by plasmid-mediated acquisition of resistance and virulence determinants (Qala Nou et al., 2025; Wang et al., 2026). In this Research Topic, one original contribution examined the molecular epidemiology, resistance profiles, clinical features, and risk factors of CR-hvKP infections in Huaian, China. Through the integration of whole-genome sequencing and comprehensive phenotypic analyses, Lian et al. delineated the population structure, resistance mechanisms, and virulence gene repertoire of circulating strains. Notably, they identified a high prevalence of blaKPC-2-producing ST11 clones, a lineage associated with rapid dissemination and severe clinical outcomes. In parallel, the study defined key host-related risk factors and clinical correlations linked to CR-hvKP infection, providing valuable epidemiological insight. Collectively, these findings deepen insight into CR-hvKP evolution and transmission while guiding surveillance, infection control, and improved therapeutic strategies.

In the original research, Sivori et al. evaluated the efficacy of meropenem/vaborbactam (MEV) against CRKP isolates belonging to the high-risk clones ST101 and ST307, recovered from catheter-related bloodstream infections. Despite harboring multidrug-resistant plasmids carrying *bla_KPC-3_* and *bla_OXA-1_*, and exhibiting resistance to several antibiotic classes, both isolates remained highly susceptible to MEV. MEV displayed potent bactericidal activity against both planktonic and biofilm-associated cells. Furthermore, MEV treatment significantly improved survival rates in the *Galleria mellonella* infection model. These results highlight MEV as a promising therapeutic option for managing complex CRKP infections, particularly those involving biofilms on medical devices.

*Escherichia coli* has become a critical-priority pathogen in the global antimicrobial resistance crisis, largely driven by the extensive use of clinically important antibiotics, which contributes to treatment failure, increased mortality, and substantial healthcare burdens worldwide (Fuga et al., 2022). This Gram-negative bacterium exhibits remarkable ecological versatility, functioning both as a commensal member of the intestinal microbiota and as an opportunistic pathogen responsible for urinary tract infections, sepsis, and other healthcare-associated diseases (Biswas et al., 2024). Furthermore, its exceptional ability to acquire resistance determinants through horizontal gene transfer complicates clinical management and accelerates the spread of multidrug-resistant strains (Rodrigues et al., 2025).

In this Research Topic, Piscon et al. aimed to evaluate the impact of the bile component glycocholic acid (GCA) on the physiology and antimicrobial susceptibility of Enterobacteriaceae such as *E. coli*. The authors demonstrated that GCA inhibits bacterial growth in liquid culture while increasing membrane permeability, leading to leakage of cytoplasmic proteins and enhanced susceptibility to antibiotics. Importantly, GCA markedly reduced bacterial motility and conjugation frequency by repressing flagellin expression and decreasing the occurrence of conjugative pili, thereby limiting horizontal transfer of resistance genes. These findings highlight GCA as a potential antimicrobial adjuvant capable of restricting bacterial growth and curbing the dissemination of antibiotic resistance.

Enteroaggregative *E. coli* (EAEC) is an emerging diarrheagenic pathotype characterized by its aggregative adherence to intestinal epithelial cells and the formation of dense biofilms that promote persistent colonization and inflammation (Ellis et al., 2020). Multidrug efflux pumps further contribute to bacterial fitness and virulence by mediating resistance to toxic compounds and facilitating host colonization (Henderson et al., 2021). In this context, Laudazzi et al. investigated the role of the AcrAB efflux system in EAEC pathogenicity. Using targeted gene deletions and functional assays, the authors demonstrated that loss of the AcrB transporter markedly impairs biofilm formation, reduces extracellular DNA export, and disrupts the characteristic aggregative adherence phenotype. Moreover, AcrB-defective strains showed attenuated virulence in a *Caenorhabditis elegans* infection model, indicating a diminished ability to colonize and damage the host. These findings identify the AcrAB pump as a key determinant linking antimicrobial resistance mechanisms with virulence traits in EAEC.

In conclusion, the studies included in this Research Topic provide new insights into the diverse mechanisms by which multidrug-resistant bacteria evade host defenses and resist antimicrobial compounds. By integrating molecular epidemiology, antimicrobial susceptibility analyses, and host–pathogen interaction studies, these contributions highlight key adaptive strategies that promote persistence, virulence, and dissemination among ESKAPE and related pathogens. Importantly, they also underscore the potential of innovative therapeutic approaches and antimicrobial adjuvants to counteract difficult-to-treat infections. Collectively, these findings advance our understanding of bacterial adaptation under antimicrobial and host-derived pressures and support the development of improved surveillance, infection control, and therapeutic strategies to combat antimicrobial resistance.
